# PSMB8 and PBK as potential gastric cancer subtype-specific biomarkers associated with prognosis

**DOI:** 10.18632/oncotarget.7411

**Published:** 2016-02-15

**Authors:** Chae Hwa Kwon, Hye Ji Park, Yu Ri Choi, Ahrong Kim, Hye Won Kim, Jin Hwa Choi, Chung Su Hwang, So Jung Lee, Chang In Choi, Tae Yong Jeon, Dae Hwan Kim, Gwang Ha Kim, Do Youn Park

**Affiliations:** ^1^ Department of Pathology, Pusan National University Hospital and Pusan National University School of Medicine, and BioMedical Research Institute, Pusan National University Hospital, Seo-Gu, Busan, Korea; ^2^ Department of Surgery, Pusan National University Hospital and Pusan National University School of Medicine, and BioMedical Research Institute, Pusan National University Hospital, Seo-Gu, Busan, Korea; ^3^ Department of Internal Medicine, Pusan National University Hospital and Pusan National University School of Medicine, and BioMedical Research Institute, Pusan National University Hospital, Seo-Gu, Busan, Korea

**Keywords:** stomach, adenocarcinoma, prognosis, PSMB8, PBK, gastric cancer

## Abstract

Gastric adenocarcinoma is a common form of cancer associated with a poor prognosis. We analyzed microarray profiling data from 48 patients with gastric adenocarcinoma to characterize gastric cancer subtypes and identify biomarkers associated with prognosis. We identified two major subtypes of gastric adenocarcinoma differentially associated with overall survival (*P* = 0.025). Genes that were differentially expressed were identified using specific criteria (*P* < 0.001 and >1.5-fold); expression of 294 and 116 genes was enriched in good and poor prognosis subtypes, respectively. Genes related to translational elongation and cell cycle were upregulated in the poor prognosis group. Of these genes, upregulation of proteasome subunit beta type 8 *PSMB8* and PDZ binding kinase *PBK* was confirmed by real-time reverse transcription-PCR analysis. *PSMB8* or *PBK* knockdown had no effect on gastric cancer cell proliferation but suppressed cell migration and invasion, respectively. Furthermore, immunohistochemistry analysis of 385 gastric cancer patients revealed that increased nuclear expression of *PSMB8* and *PBK* was correlated with depth of invasion, lymph node metastasis, and lower survival rates. Taken together, two gastric adenocarcinoma subtypes were predictive of prognosis. *PSMB8* and *PBK* were predictive of gastric cancer prognosis and could be potential gastric cancer subtype-specific biomarkers.

## INTRODUCTION

Gastric cancer is a common form of cancer with the second highest cancer-related mortality rate [1]. Advanced gastric cancer is generally refractory to chemotherapy, leading to a poor prognosis, with five-year survival rates of only 20-30% [2]. Even in patients at the early stage of the disease, the presence of lymph node metastasis considerably decreases survival rates [3]. Although the histological and pathological stages of gastric cancer have been the gold standard for determining prognosis, these only offer limited information about disease status in individual patients.

There is heterogeneity among tumors displaying a similar histopathological appearance, which results in different clinical outcomes. Thus, it is important to understand the molecular heterogeneity of tumors and to identify clinically useful biomarkers to identify gastric cancer patients with a poor prognosis for alternative treatment strategies. Biomarkers could advance the development of diagnostic or prognostic systems and anti-cancer drugs, while also providing more comprehensive information relating to mechanisms underlying tumor-related processes. Previous studies have been focused on discovering novel biomarkers or gene signatures associated with gastric cancer using gene expression profiling [4, 5]. However, the subjective supervised methods used in these studies were suggested to result in bias relating to relevant processes, leading to classification that is not biologically meaningful [6]. Therefore, in the present study, we analyzed gene expression profiling data from 48 patients with gastric cancer and identified two subtypes that were clinically relevant in predicting prognosis based on their unique gene expression signatures using an unsupervised hierarchical clustering. In addition, we identified and validated two specific biomarkers associated with prognosis.

## RESULTS

### Two subgroups of gastric cancer associated with prognosis

**Figure 1:**
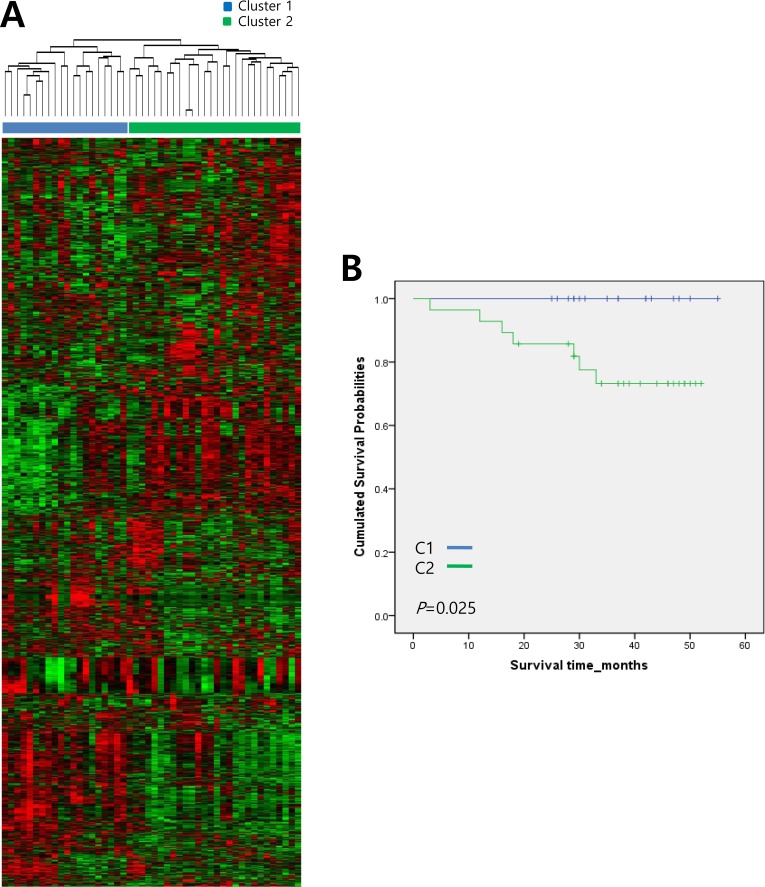
Hierarchical clustering analysis of gene expression data from 48 human gastric adenocarcinoma tissue samples **A.** Genes with expression levels that were at least two-fold different in at least ten tissue samples were selected for unsupervised hierarchical clustering analysis (2,800 gene features). The results show two distinctive subtypes with significant differences in their respective gene expression signatures. Data are presented in a matrix format, in which each row represents an individual gene and each column represents a different tissue sample. Each cell in the matrix represents the expression level of a gene feature in an individual tissue sample. Red, high expression; green, low expression. **B.** Kaplan-Meier survival curves of the two clusters based on gene expression signature. *P*-values were obtained using the log-rank test.

An unsupervised hierarchical clustering analysis was performed to evaluate gene expression data obtained from 48 human gastric adenocarcinoma tissue samples (Supplementary Table 1). Genes with expression levels that displayed at least a two-fold difference in a minimum of ten tissue samples were selected for hierarchical clustering analysis (2,800 genes total). Unsupervised hierarchical clustering revealed two distinctive subtypes (Cluster 1 (C1, n = 20) and Cluster 2 (C2, n = 28)) with clear differences in gene expression profiles (Figure 1A). Two subtypes did not correlate with level of tumor differentiation or histological type of tumor (data not shown). To further evaluate the association of these two subtypes with prognosis, we performed a Kaplan-Meier survival analysis. As shown in Figure 1B, C2 patients had a significantly lower overall survival rate compared with that of C1 patients (*P* = 0.02, log-rank test). The average survival durations of the C1 and C2 subtypes were 24.7 and 23.7 months, respectively. These results suggest that the molecular signatures of these gastric tumors could be useful predictors of clinical outcomes.

### Gene expression signatures in gastric cancer associated with prognosis

**Figure 2:**
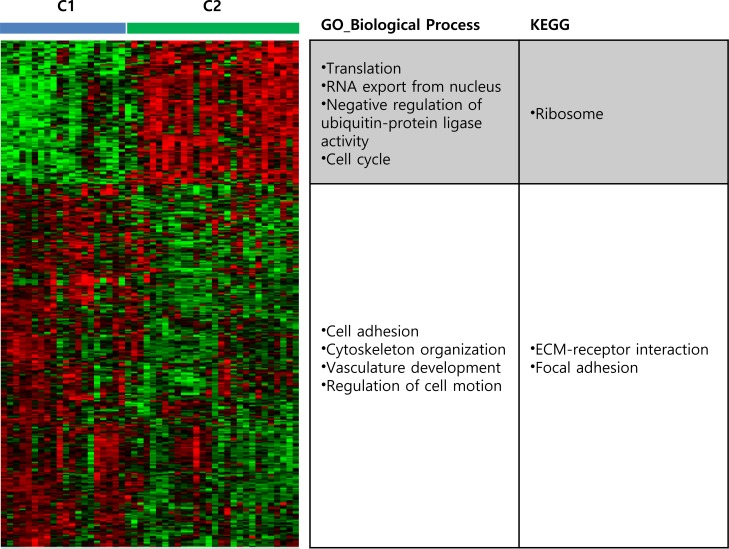
Subtype-specific gene signature and annotation Two-sample *t*-tests were applied to gene expression data from the two subtypes and differences were considered statistically significant if *P* < 0.001 and >1.5 fold change. Differentially expressed genes were categorized based on biological processing and KEGG pathway. Red, high expression; green, low expression.

We next sought to identify genes that were differentially expressed between C1 and C2 subtypes by applying the following thresholds: *P* < 0.001 and >1.5 fold change. We identified 294 and 116 genes displaying higher expression in C1 and C2 subtypes, respectively. We analyzed these gene lists using DAVID functional annotation tools to categorize the enriched genes into subtypes based on biological process (BP) and KEGG pathway (Figure 2 and Supplementary Table 2). Genes highly expressed in the C1 subtype were related to the following gene sets: GO_BP cell adhesion (*P* = 8.62 × 10^−18^), cytoskeleton organization (*P* = 5.36 × 10^−6^), regulation of cell motion (*P* = 4.66 × 10^−6^), and vasculature development (*P* = 1.75 10^−5^); KEGG ECM-receptor interaction (*P* = 2.44 × 10^−9^), focal adhesion (*P* = 2.61 × 10^−8^), and vascular smooth muscle contraction (*P* = 3.66 × 10^−5^).

Genes more highly expressed in the C2 subtype were related to the following genes sets: GO_BP translation (*P* = 5.64 × 10^−4^), RNA export from nucleus (*P* = 2.93 × 10^−5^), negative regulation of ubiquitin-protein ligase activity during mitotic cell cycle (*P* = 2.84 × 10^−3^), cell cycle (*P* = 1.88 × 10^−2^), and KEGG ribosome (*P* = 2.13 × 10^−4^). Of the 116 genes highly expressed in the C2 subtype, 22 were associated with these GO terms and KEGG pathways, most of which are implicated in cancer: Ribosomal protein (RP)-encoding genes *RPL6*, *RPLP0*, *RPL8*, *RPS6P1*, *PRS7*, and *RPL29* [9], eukaryotic translation elongation factor *EEF1B2* [10], eukaryotic translation initiation factor *EIF5A* [11], mRNA export factor *THOC4* [12], heterogeneous nuclear ribonucleoprotein *HNRNPA1* [13], mitotic arrest deficiency 2 *MAD2* [14], proteasome subunits *PSMA6* and *PSMB8* [15], protein kinase regulatory subunit *CKS2* [16], and PDZ-binding kinase *PBK* [17]. These results suggest that altered expression of these identified genes could reflect the specific molecular and biological features of gastric cancer cells.

### Validation of gene expression

To validate the candidate genes that were highly expressed in C2 patient samples with poor prognosis, we performed a real-time PCR analysis using the same RNA used for the microarray analysis. The C_T_ values for *THOC4*, *PSMB8*, *CKS2* and *PBK* were significantly lower in C2 than C1, suggesting increased expression of these genes was associated with a poor prognosis (Supplementary Figure 1). These results are consistent with data from our microarray analysis.

We focused on *PSMB8* and *PBK*, whose functions in gastric cancer have not been reported. The Oncomine database (http://www.oncomine.org) was used to examine the differences in mRNA levels of *PSMB8* and *PBK* between gastric cancer tissues and adjacent normal tissues. As shown in Supplementary Figure 2A and 2B, *PSMB8* and *PBK* in microarray datasets (Cho *et al*. [18], and D'Errico *et al*. [19]) were overexpressed in gastric cancer tissues compared with that in normal tissues (*P* = 0.006, *P* = 0.007, *P* = 0.001, *P* = 4.34E-9). In addition, the protein levels of these molecules in gastric cancers were significantly higher than those in adjacent normal tissue, as shown in Supplementary Figure 2C and 2D.

### Roles of PSMB8 and PBK in gastric cancer cells

**Figure 3:**
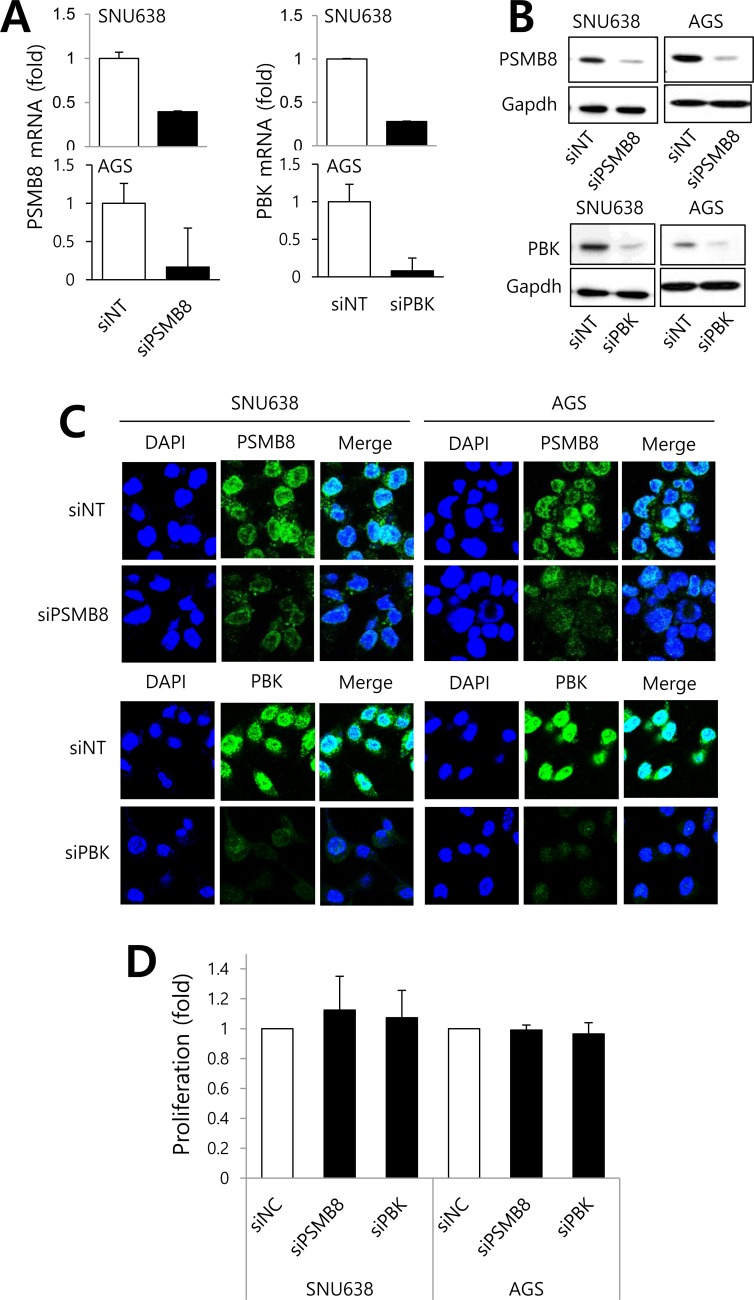
The roles of PSMB8 and PBK in proliferation of gastric cancer SNU638 and AGS cells were transfected with either non-targeting siRNA (siNT), *PSMB8* siRNA (siPSMB8), or *PBK* siRNA (siPBK) for 48 hours. PSMB8 and PBK mRNA and protein expression was determined using real-time PCR **A.**, western blotting **B.**, and immunocytochemical analysis **C. D.** After transfection, proliferation was evaluated using an MTT assay.

**Figure 4:**
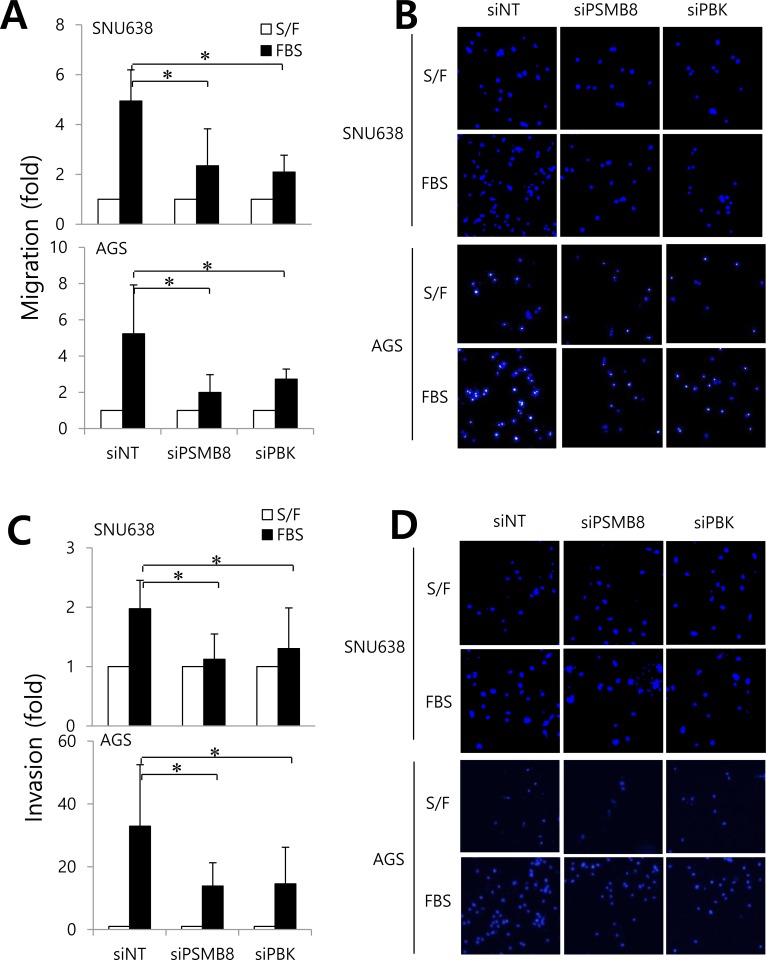
The roles of PSMB8 and PBK in migration and invasion of gastric cancer SNU638 and AGS cells were transfected with either non-targeting siRNA (siNT), *PSMB8* siRNA (siPSMB8), or *PBK* siRNA (siPBK) for 48 hours. The migration and invasion assays were subsequently performed. Graphs and representative data show cells that migrated **A.** and **B.** and invaded (C and D) in the presence (S/F, serum free) or absence of 1% (FBS). *P < 0.05.

To investigate the roles of *PSMB8* and *PBK* in gastric cancer cells, SNU638 and AGS cells expressing high levels of these genes (Supplementary Figure 3), were transfected with siRNAs specific for each gene, and proliferation, migration, and invasion assays were performed. Gene knockdown was verified using real-time PCR, western blotting, and immunocytochemistry (Figure 3A-3C). Knockdown of either *PSMB8* or *PBK* had no effect on gastric cancer cell proliferation (Figure 3D). However, knockdown of each gene significantly decreased gastric cancer cell migration and invasion (Figure 4A-4D), suggesting that PSMB8 and PBK are involved in gastric cancer progression.

### Clinical relevance of PSMB8 and PBK expression in gastric cancer cells

**Figure 5:**
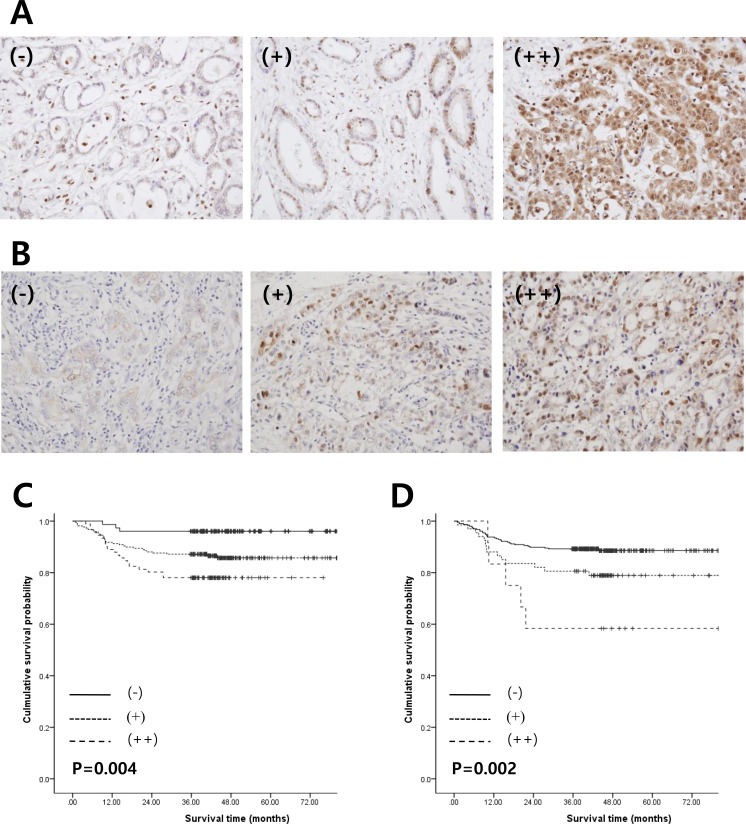
Relationship between expression of PSMB8 or PBK and clinical outcomes in patients with gastric cancer **A.** and **B.** PSMB8 and PBK protein expression levels were determined by immunohistochemistry analysis. (−), (+) or (++) nuclear staining for PSMB8 or PBK was observed in tumor cells. **C.** and **D.** Kaplan-Meier survival analysis was performed according to PSMB8 or PBK expression. *P*-values were calculated using the log-rank test.

We next performed immunohistochemistry staining to detect PSMB8 and PBK protein expression in 385 gastric cancer specimens that were not the same as those used in the microarray studies. PSMB8 expression was identified in the cytoplasm and nuclei of gastric adenocarcinoma cells (Figure 5A). Regarding nuclear PSMB8 expression, positive staining at levels of either < 5% (−) or 5-25% (+), and > 25% (++) was observed in 62.3% (249 of 385) and 23.6% (91 of 385) of cases, respectively. Regarding cytoplasmic PSMB8 expression, - (240/385, 62.3%), + (104/285, 27.0%), and ++ (41/385, 10.6%) were observed in gastric adenocarcinoma cells.

We investigated the clinicopathological and prognostic significance of nuclear PSMB8 expression in 385 cases of gastric adenocarcinoma (Table 1). Nuclear PSMB8 expression was associated with increased invasiveness of gastric adenocarcinoma, including higher stage (*P* < 0.0001), depth of invasion (*P* = 0.003), lymph node metastasis (*P* < 0.0001), lymphovascular tumor emboli (*P* < 0.0001), increased tumor size (*P* = 0.002), and perineural invasion of tumor cells (*P* = 0.040). However, no significant differences in nuclear PSMB8 expression were associated with patient sex, age, or location. Consistent with the association between nuclear PSMB8 expression and gastric adenocarcinoma aggressiveness, the patient group survival rate decreased as nuclear PSMB8 expression increased (Figure 5C). The average survival durations of the (−), (+), and (++) groups, which were classified according to nuclear PSMB8 expression, were 77.2, 70.6, and 62.2 months, respectively; this decrease was statistically significant (*P* = 0.004). However, cytoplasmic PSMB8 expression was not associated with clinicopathological variables or poor outcomes (data not shown).

**Table 1:** Relationship between nuclear PSMB8 expression and clinicopathological characteristics in 385 patients with gastric cancer

	[No.]	Nuclear PSMB8 expression		*P* value
		(−) (+)	(++)	
Age (years)	385	59.6 ± 0.66	61.2 ± 1.18	0.224
Size (cm)	385	4.52± 0.16	5.65 ± 0.38	0.002
Sex				
Male	247	187 (75.7)	60 (24.3)	0.686
Female	138	107 (77.5)	31 (22.5)	
Location				
Upper	43	30 (69.8)	13 (30.2)	0.502
Middle	126	99 (78.6)	27 (21.4)	
Lower	216	165 (76.4)	51 (23.6)	
Gross type¶				0.011
Elevated	88	65 (73.9)	23 (26.1)	
Flat/Depressed	143	121 (84.6)	22 (15.4)	
Excavated	154	108 (70.1)	46 (29.9)	
Histological type*				0.443
Intestinal	219	164 (74.9)	55 (25.1)	
Diffuse	162	127 (78.4)	35 (21.6)	
Mixed	4	3 (75.0)	1 (25.0)	
Stage				<0.0001
I	196	166 (84.7)	30 (15.3)	
II	74	57 (77.0)	17 (23.0)	
III	115	71 (61.7)	44 (38.3)	
Invasion depth				0.003
T1	181	150 (82.9)	31 (17.1)	
T2	59	48 (81.4)	11 (18.6)	
T3	62	43 (69.4)	19 (30.6)	
T4	83	53 (63.9)	30 (36.1)	
Perineural invasion				0.040
Negative	226	181 (80.1)	45 (19.9)	
Positive	159	113 (71.1)	46 (28.9)	
Lymphovascular emboli				<0.0001
Negative	236	196 (83.1)	40 (16.9)	
Positive	149	98 (65.8)	51 (34.2)	
Lymph node metastasis‡				<0.0001
N0	205	173 (84.4)	32 (15.6)	
N1	53	42 (79.2)	11 (20.8)	
N2	60	38 (63.3)	22 (36.7)	
N3	67	41 (61.2)	26 (38.8)	

¶ between elevated+flat/depressed vs excavated

* intestinal+mixed vs diffuse

PBK expression was identified in cytoplasm and nuclei of gastric adenocarcinoma cells (Figure 5B). Nuclear/cytoplasmic PBK expression in gastric adenocarcinoma tumor cells was classified as follows: - (306/385, 79.5%), + (67/385, 17.40%), and ++ cytoplasmic expression (12/385, 3.1%); - (234/385, 60.8%), + (117/285, 30.4%), and ++ nuclear expression (34/385, 8.8%). Nuclear PBK expression was associated with increased gastric adenocarcinoma invasiveness, including higher stage (*P* = 0.002), depth of invasion (*P* = 0.017), and lymph node metastasis (*P =* 0.001), similar to the PSMB nuclear expression results. However, no significant differences in nuclear PSMB8 expression status were associated with patient sex, age, location, perineural invasion, or lymphovascular tumor emboli (Table 2). Increased nuclear PBK expression was associated with a poor prognosis in gastric adenocarcinoma ((−), 72.3 ± 1.2; (+), 66.2 ± 3.4; (++), 53.1 ± 9.2; *P* = 0.002) (Figure 5D). However, cytoplasmic PBK expression was not associated with increased gastric adenocarcinoma invasiveness or poor outcomes (data not shown).

**Table 2:** Relationship between nuclear PBK expression and clinicopathological characteristics in 385 patients with gastric cancer

		Nuclear PBK/TOPK expression		*P*value
	[No.]	(−)	(+) (++)	
Age (years)	385	59.7 ± 0.65	60.8 ± 1.20	0.426
Size (cm)	385	4.58± 0.17	5.57 ± 0.37	0.010
Sex				
Male	247	192 (77.7)	55 (22.3)	0.256
Female	138	114 (82.6)	24 (17.4)	
Location				
Upper	43	32 (74.4)	11 (25.6)	0.489
Middle	126	98 (77.8)	28 (22.2)	
Lower	216	176 (81.5)	40 (18.5)	
Gross type				0.515
Elevated	88	72 (80.7)	27 (19.3)	
Flat/Depressed	143	117 (81.8)	26 (18.2)	
Excavated	154	118 (76.6)	36 (23.4)	
Histological type*				0.208
Intestinal	219	179 (81.7)	40(18.3)	
Diffuse	162	123 (75.9)	39 (24.1)	
Mixed	4	4 (100.0)	0 (0.0)	
Stage				0.002
I	196	169 (86.2)	27 (13.8)	
II	74	57 (77.0)	17 (23.0)	
III	115	80 (69.6)	35 (30.4)	
Invasion depth				0.017
T1	181	153 (84.5)	28 (15.5)	
T2	59	50 (84.7)	9 (15.3)	
T3	62	44 (71.0)	18 (29.0)	
T4	83	59 (71.1)	24 (28.9)	
Perineural invasion				0.102
Negative	226	186 (82.3)	40 (17.7)	
Positive	159	120 (75.5)	39 (24.5)	
Lymphovascular emboli				0.054
Negative	236	195 (82.6)	41 (17.4)	
Positive	149	98 (74.5)	51 (25.5)	
Lymph node metastasis‡				0.010
N0	205	175 (85.4)	30 (14.6)	
N1	53	40 (75.5)	13 (24.5)	
N2	60	46 (76.7)	14 (23.3)	
N3	67	45 (67.2)	22 (32.8)	

* intestinal+mixed vs diffuse

In addition, multivariate analysis showed that nuclear expression of PSMB8 and PBK were borderline significant predictors of overall survival (*P* = 0.085, *P* = 0.083, respectively) (Supplementary Table 3 and 4). These findings indicate that nuclear PSMB8 and PBK overexpression in tumor cells correlates with gastric cancer progression, especially aspects relating to tumor invasion depth and lymph node metastasis. These genes could be candidate biomarkers for prediction of gastric cancer survival.

## DISCUSSION

We performed microarray-based profiling to identify subtypes of gastric adenocarcinoma with different gene expression signatures. Using an unsupervised clustering approach, we found that gastric cancer patients were divided into two distinct subtypes that significantly differed with respect to overall survival, underscoring the clinical relevance of these subtypes. In addition, we identified 410 genes that could predict the prognosis of gastric cancer patients. Of these 410 genes, 294 were related to cell adhesion and ECM and were highly expressed in the subtype with a good prognosis. Previous studies have reported that inhibition of collagen fibril formation increases tumor cell invasion and ECM accumulation, contributing to tumor cell necrosis [20, 21]. These findings indicate that ECM constituents inhibit tumor progression, resulting in a good prognosis. Of 410 genes, 116 were highly expressed in the subtype with a poor prognosis. The biological functions of these 116 genes were associated with cancer and included translation, RNA transport, cell cycle, and the ribosome pathway.

Consistent with our results, a previous study identified ribosomal proteins as prognostic markers for gastric cancer [6]. Dysregulation of ribosome synthesis promotes tumor formation [22]. Ribosome synthesis and protein translation are closely coordinated processes [23]. Translation elongation factor *EEF1B2* and translation initiation factor *EIF5A* have non-canonical functions unrelated to protein synthesis [24], which are reported to control cancer cell proliferation [10, 25]. Prior to translation, mRNA is processed, edited, and exported. We found that *THOC4* and *HNRNPA1*, which are associated with mRNA processing, were overexpressed in the C2 subtype with a shorter survival period. These genes have been reported to display altered expression in various forms of cancer, with higher expression associated with cancer development [12, 13, 26, 27].

Genes related to the cell cycle were also identified as upregulated in tissue from patients with a poor prognosis. *MAD2* and *BUB3* are involved in spindle checkpoint function, with increased *MAD2* expression associated with the formation of aggressive tumors in multiple organs [14]. *PBK* is a serine/threonine protein kinase related to the mitogen-activated protein kinase kinase (MAPKK) family. Overexpression of this gene has been implicated in tumorigenesis [17, 28]. Moreover, the cyclin kinase subunit *CKS2* was upregulated; *CKS2* expression was increased in several types of cancer and associated with tumor progression [29, 30]. Tumor cells proliferate rapidly and have high rates of protein synthesis, consequently displaying higher proteasome activity [31, 32]. Consistent with this, proteasome subunit PSMA6 and PSMB8 were more highly expressed in gastric cancer patients with a poor prognosis compared with patients with a good prognosis.

Because the C2 subtype was highly associated with a poor prognosis, we validated genes that were highly expressed in the C2 subtype using real-time PCR. *THOC4*, *PSMB8*, *CKS2,* and *PBK* were validated. Of these genes, we focused on *PSMB8* and *PBK*. The proteasome system is a non-lysosomal proteolytic pathway with important roles in many cellular processes, such as cell cycle regulation, proliferation, differentiation, and inflammation [33]. Other proteasome subunits have been shown to be overexpressed in some tumor cell types [34, 35]. However, the role of *PSMB8*, a component of the 20S proteolytic core particle of the proteasome, has not been reported in cancer. Although *PBK* is also involved in many cellular functions, including tumor development, cell growth and cell death, and is highly expressed in many cancers [36-39], its role in gastric cancer has not been reported. Thus, we investigated the functional role of these genes on gastric cancer using siRNAs against each gene. Knockdown of *PSMB8* and *PBK* decreased gastric cancer cell migration and invasion, respectively.

We next performed an immunohistochemistry analysis of 385 gastric cancer patients to evaluate the relationship between expression of PSMB8 or PBK and clinicopathological characteristics. Strong nuclear expression of PSMB8 and PBK significantly correlated with increased depth of invasion and lymph node metastasis, respectively. Elevated nuclear expression of PSMB8 was related to lymphovascular tumor emboli, increased tumor size, and perineural invasion of tumor cells. Consistent with our microarray results, a Kaplan-Meier survival analysis revealed that gastric cancer patients displaying high protein expression of either PSMB8 or PBK had a decreased survival rate. These results indicate that PSMB8 and PBK promote carcinogenesis and gastric cancer metastasis, and are potential biomarkers able to predict a poor prognosis. In addition, PSMB8 and PBK are useful immunohistochemical protein markers for potential surgical pathology application, as prediction of lymph node metastasis based on biopsy specimen evaluation prior to endoscopic resection is a critical decision point. Other studies described gene signatures associated with gastric cancer prognosis. These approaches cannot be easily translated into the clinic due to of the difficulty of acquiring fresh-frozen tissues from patients and the complexity of data analysis [18]. To our knowledge, this is the first report to characterize the roles of PSMB8 and PBK in gastric cancer progression.

Although we clearly suggested that PSMB8 and PBK promote the migration and invasion of gastric cancer cells, PSMB8 and PBK are not sufficient to proliferation by themselves in gastric cancer cells. Additional factor might be required to cooperate in inducing proliferative effect of PSMB8 or PBK. The molecular mechanism by which PSMB8 and PBK promote gastric cancer cell progression has not been elucidated. Although it has been reported that carcinoma cell motility and invasiveness occur via the PI3K [40] and MAP kinase pathways [41], we observed unaltered activation of Akt, ERK, or p38 following PSMB8 or PBK downregulation in gastric cancer (Supplementary Figure 4). These findings suggest that activation of the PI3K or MAPK pathways was not involved in PSMB8 or PBK-induced cell motility and invasiveness. Proteasome subunit has been reported to regulate transcription factors by nuclear localization and promoter interaction [42]. Another subunit, PSMB1, resides in the nucleus and binds to the PAI-2 and Reg1 promoters to upregulate expression of target genes related to tumor progression [43]. PBK is also localized to nuclei, where it is involved in phosphorylation of histone H3 and inhibition of p53 in colorectal cancer and breast cancer cells, respectively [37, 44]. In addition, PBK is reported to correlate with mutant p53 and affects cell proliferation and viability in lung adenocarcinoma [45]. In future studies, we will investigate the underlying mechanisms of PSMB8 and PBK in detail.

In conclusion, we identified the expression signatures of two distinct subtypes of gastric cancer associated with different survival rates. Further validation of these gene signatures will be necessary in a larger cohort of patients. We propose that PSMB8 and PBK could be useful biomarkers for identifying gastric cancer patients with a poor prognosis. These findings could contribute to developing an improved method of molecular classification of gastric cancer patients that can predict survival. These markers also have the potential for clinical application, including the development of diagnostic markers and therapeutic agents for gastric cancer.

## MATERIALS AND METHODS

### Patients and samples

As we described in our previous study [7], a total of 48 fresh gastric cancer tissues were obtained with informed consent from patients who underwent gastrectomy at Pusan National University Hospital (PNUH) and Cheon Nam National University Hospital (CNUH), members of the National Biobank of Korea, which is supported by the Ministry of Health, Welfare, and Family Affairs, and were analyzed by microarray. Clinical characteristics of the patients were shown in Supplementary Table 1.

### Gene expression data and analysis

Total RNA was extracted from 48 fresh-frozen tissues using a mirVana RNA Isolation kit (Ambion Inc., Austin, TX). Five-hundred nanograms of total RNA was used for cDNA synthesis, which was followed by an amplification/labeling step (*in vitro* transcription) using the Illumina TotalPrep RNA Amplification kit (Ambion). Labeled, amplified material (1500 ng per array) was hybridized to Illumina HumanHT-12 BeadChips v4.0, according to the manufacturer's instructions (Illumina, San Diego, CA). Array signals were developed by streptavidin-Cy3. Arrays were scanned with an Illumina iScan system. The microarray data were normalized using the quantile normalization method in Illumina BeadStudio software. Microarray data are available in NCBI's GEO (Gene Expression Omnibus) database (http://www.ncbi.nlm.nih.gov/geo/query/acc.cgi?acc=GSE38024).

The expression level of each gene was transformed into a log^2^ base before further analysis. Excel was primarily used for the statistical analyses. Gene expression differences were considered statistically significant if the *P* value was <0.001 and all tests were 2-tailed. Cluster analyses were performed using Cluster and Treeview. The gene ontology (GO) program (http://david.abcc.ncifcrf.gov/) was used to categorize genes into subtypes based on biological function and pathway. Fisher's exact test was used to determine whether the proportions of genes in each category differed by group. Prognostic significance was estimated by Kaplan-Meier plots and log-rank tests between two predicted subtypes of patients. This statistical calculation was performed with SPSS version 10.0 for Windows (SPSS Inc., Chicago, IL, USA).

### Real-time PCR

Total RNA was extracted from gastric cancer cells, using the TRIzol reagent (Invitrogen), following the manufacturer's instructions. RNA was reverse transcribed with SuperScript II (Invitrogen) and cDNA was amplified with each primer and visualized with SYBR Green (Applied Biosystems; Life Technologies; NY, USA), using the fluorescence reader Corbett Rotor-Gene 6000 (Qiagen Inc., CA, USA). The primers used are the following:: glyceraldehyde 3-phosphate dehydrogenase (GAPDH), 5′-TCCATGACAACTTTGGTATCG-3′, 5′-TGTAGCCAAATTCGTTGTCA-3′; PSMB8, 5′-GCTGCCTTCAACATAACATCA-3′, 5′-CTGCCACCACCACCATTA-5′; CKS2, 5′-TAA GGCAACTGGTAAGCATTC-3′, 5′-ACAAGATACAGCCAAGTGTTAGTCC-3′; PBK, 5′- GCCAGCCAAGATCCTTTTCC-3′, 5′- TCTGTGACGTGACAAGCTGA-5′; THOC4, 5′- TTTGGAACGCTGAAGAAGGC-3′, 5′- TCTGTGACGTGACAAGCTGA-3′ The following thermal cycler program was used: denaturation for 30 s at 95°C; annealing for 30 s at 52°C, depending on the primers used; and extension for 30 s at 72°C. The number of PCR cycles was determined for each gene and ranged from 25 to 35. Data were normalized to GAPDH, and mRNA abundance was calculated using the 2^−ΔΔCT^ method.

### Cell lines and transfection

The human gastric cancer cell lines SNU638 was obtained from the Korean Cell Line Bank (Seoul, South Korea) and were authenticated. These cells were cultured in RPMI1640 medium with 10% fetal bovine serum (FBS; GIBCO; Thermo Scientific Inc.; PA, USA), 100 U/mL penicillin, and 100 μg/mL streptomycin (Sigma-Aldrich; MO, USA). All cells were maintained at 37°C in 5% CO_2_. For knockdown of genes, cells were transfected with PSMB8 or PBK smartpool short interfering RNA (siRNA) or with non-targeting siRNA as a control (Dharmacon; Thermo Scientific Inc.; PA, USA), using Lipofectamine RNAiMAX reagent (Invitrogen) according to the manufacturer's instructions. The siRNA sequences were as follows: PSMB8 (5696) siRNA, 5′-UGAUUGAGAUUAACCCUUA-3′, 5′-UCAGCUGGGUCCUACAUUA-3′, 5′-GGCUAUCGGCCUAAUCUUA-3′, 5′-GAGAACGUAUUUCAGUGUC-3′; PBK (55872) siRNA, 5′-CAAGACACCAAGCAAAUUA-3′, 5′-GGCAAGAGGGUUAAAGUAU-3′, 5′-GUUCAACUCCAACUAUAAA-3′, 5′-GAUCAUUAUCGAAGUGUGU-3′.

### Cell proliferation

Cell proliferation was evaluated using MTT assay. Cells were transfected with siRNA for 48 hours and then washed. Culture medium containing 0.5 mg/ml of MTT was added to each well. The cells were incubated for 3 h at 37°C, the supernatant was removed and the formed formazan crystals in viable cells were solubilized with dimethyl sulfoxide. A 0.1 ml aliquot of each sample was then translated to 96-well plates and the absorbance of each well was measured at 550 nm with spectrophotometer.

### Immunocytochemistry

Cells were cultured on glass coverslips and transfected with siRNA. Cells were washed twice with PBS, fixed with 4% paraformadehyde in PBS for 10 min, permeabilized with 0.5% Triton X-100 in PBS for 10 min. After washing twice with PBS, cells were blocked with 8% BSA in Tris-buffered saline Triton X-100 (TBST). Cells were incubated with anti-PSMB8 or anti-PBK overnight 4°C and washed twice with TBST. Cells were incubated with FITC-conjugated secondary antibody (Jackson ImmunoResearch Laboratories, PA, USA) for 1 h, and the nuclei were counterstained with DAPI to determine nuclear localization. Coverslips were mounted and visualized by using the confocal microscope.

### Cell migration and invasion assays

Gastric cancer cells were harvested with 0.05% trypsin containing 0.02% EDTA (Sigma-Aldrich) and suspended in RPMI medium. For the migration assay, membrane filters (8-μm pore size) in disposable 96-well chemotaxis chambers (Neuro Probe; Gaithersburg, MD, USA) were pre-coated with 5 mg/mL fibronectin for 4 h at room temperature. Cells (3 × 10^3^ cells/well) were loaded into the upper chambers, and 1% FBS was loaded into the lower chamber. After 24 h of incubation, non-migrating cells were removed from the upper chamber with a cotton swab, and the cells on the lower surface of the insert were stained with Hoechst33342 (Sigma-Aldrich). Migrated cells were counted under a fluorescence microscope at 10× magnification.

For the invasion assay, 3 × 10^4^ cells/well were seeded in the upper chamber, which was coated with 30 μL of Matrigel (1 mg/mL in cold medium; BD Transduction Laboratories; NJ, USA). Serum-free medium containing 1% FBS or control vehicle was added to the lower chamber. After 24 h of incubation, non-invading cells were removed from the upper chamber with a cotton swab, and cells on the lower surface of the insert were stained with Hoechst33342 (Sigma-Aldrich). Invasive cells were counted under a fluorescence microscope at 10× magnification.

### Western blot analysis

Cells were harvested and disrupted in lysis buffer (1% Triton X-100, 1 mM EGTA, 1 mM EDTA, 10 mM Tris-HCl at pH 7.4, and protease inhibitors). Cell debris was removed via centrifugation at 10,000 × *g* for 10 min at 4°C. The resulting supernatants were resolved using SDS-PAGE and transferred onto nitrocellulose membranes. The membranes were blocked with 5% non-fat dried milk at room temperature for 30 min and incubated with anti-PSMB8 (Abcam; MA, USA), anti-PBK, anti-Akt, anti-ERK, anti-p38 (Cell Signaling Technology; MA, USA), and anti-GAPDH. The membranes were then washed and incubated with horseradish peroxidase-conjugated secondary antibody. Signals were visualized using enhanced chemiluminescence (Amersham; Buckinghamshire, UK).

### Immunohistochemistry and analysis of clinicopathological and prognostic significance

We studied a cohort of 385 gastric cancer patients who underwent gastrectomy with lymph node dissection at PNUH between 2005 and 2007. None of the patients received preoperative radiotherapy or chemotherapy. Standard formalin-fixed and paraffin-embedded sections were obtained from the Department of Pathology and the National Biobank of Korea, PNUH. All samples from the National Biobank of Korea were obtained with informed consent under institutional review board-approved protocols.

Methods of immunohistochemistry have previously been described [8]. The percentage of positive cells showing moderate to strong staining intensity was scored. The score is explained as follows: (−), < 5%; (+), 5%-25%; (++), > 25%. Clinicopathological features were analyzed for differences in PSMB8 or PBK expression using the Student's *t*-test, the χ^2^ test, or Fisher's exact test. The relationships between expression of PSMB8 and PBK were assessed with a Spearman rank correlation coefficient. Cumulative survival plots were obtained using the Kaplan-Meier method, and significance was compared using the log-rank test. Statistical significance was set at *P* < 0.05. Multivariate analyses were carried out using Cox proportional hazards regression. Statistical calculations were performed using SSPS version 10.0 for Windows (SPSS Inc.; Chicago, IL, USA).

## SUPPLEMENTARY MATERIAL FIGURES AND TABLES



## References

[2] Parkin DM, Bray F, Ferlay J, Pisani P (2005). Global cancer statistics 2002. CA Cancer J Clin.

[2] De Vita F, Giuliani F, Galizia G, Belli C, Aurilio G, Santabarbara G, Ciardiello F, Catalano G, Orditura M (2007). Neo-adjuvant and adjuvant chemotherapy of gastric cancer. Ann Oncol.

[3] Zhang XF, Huang CM, Lu HS, Wu XY, Wang C, Guang GX, Zhang JZ, Zheng CH (2004). Surgical treatment and prognosis of gastric cancer in 2,613 patients. World J Gstroenterol.

[4] Cui J, Li F, Wang G, Fang X, Puett JD, Xu Y (2011). Gene-expression signatures can distinguish gastric cancer grades and stages. PLoS One.

[5] Cho JY, Lim JY, Cheong JH, Park YY, Yoon SL, Kim SM, Kim SB, Kim H, Hong SW, Park YN, Noh SH, Park ES, Chu IS, Hong WK, Ajani JA, Lee JS (2011). Gene expression signature-based prognostic risk score in gastric cancer. Clin Cancer Res.

[6] Zhang Y, Zhang L, Gao Y, Li C, Jia S, Liu N, Chen F, Niu D, Cho WC, Ji J, Zeng C (2011). Discovery and validation of prognostric markers in gastric cancer by genome- wide expression profiling. World J Gastroenterol.

[7] Shin NR, Jeong EH, Choi CI, Moon HJ, Kwon CH, Chu IS, Kim GH, Jeon TY, Kim DH, Lee JH, Park do Y (2012). Overexpression of Snail is associated with lymph node metastasis and poor prognosis in patients with gastric cancer. BMC Cancer.

[8] Kwon CH, Park HJ, Lee JR, Kim HK, Jeon TY, Jo HJ, Kim DH, Kim GH, Park DY (2014). Serpin peptidase inhibitor clade A member 1 is a biomarker of poor prognosis in gastric cancer. Br J Cancer.

[9] Lai MD, Xu J (2007). Ribosomal proteins and colorectal cancer. Curr Genomics.

[10] Caraglia M, Budillon A, Vitale G, Lupoli G, Tagliaferro P, Abruzzese A (2000). Modulation of molecular mechanisms involved in protein synthesis machinery as a new tool for control of cell proliferation. Eur J Biochem.

[11] Byun HO, Han NK, Lee HJ, Kim KB, Ko YG, Yoon G, Lee YS, Hong SI, Lee JS (2009). Cathepsin D and eukaryotic translation elongation factor 1 as promising markers of cellular senescence. Cancer Res.

[12] Domínguez-Sánchez MS, Sáez C, Japón MA, Aguilera A, Luna R (2011). Differential expression of THOC1 and ALY mRNP biogenesis/export factors in human cancers. BMC cancer.

[13] Ma YL, Peng JY, Zhang P, Huang L, Liu WJ, Shen TY, Chen HQ, Zhou YK, Zhang M, Chu ZX, Qin HL (2009). Heterogeneous nuclear ribonucleoprotein A1 is identified as a potential biomarker for colorectal cancer based on differential proteomics technology. J Proteome Res.

[14] Schvartzman JM, Sotillo R, Benezra R (2010). Mitotic chromosomal instability and cancer: Mouse modelling of the human disease. Nat Rev Cancer.

[15] Bhui-Kaur A, Therwath A, Henry L, Chiesa J, Kurkure A, Scherrer K, Bureau JP (1998). Increased prosomal proteins in breast cancer cells and in neighboring normal cells in Parsi and non-Parsi populations. J Cancer Res Clin Oncol.

[16] Tanaka F, Matsuzaki S, Mimori K, Kita Y, Inoue H, Mori M (2011). Clinicopathological and biological significance of CDC28 protein kinase regulatory subunit 2 overexpression in human gastric cancer. Int J Oncol.

[17] Shih MC, Chen JY, Wu YC, Jan YH, Yang BM, Lu PJ, Cheng HC, Huang MS, Yang CJ, Hsiao M, Lai JM (2012). TOPK/PBK promotes cell migration via modulation of the PI3K/PTEN/AKT pathway and is associated with poor prognosis in lung cancer. Oncogene.

[18] Cho JY, Lim JY, Cheong JH, Park YY, Yoon SL, Kim SM, Kim SB, Kim H, Hong SW, Park YN, Noh SH, Park ES, Chu IS, Hong WK, Ajani JA, Lee JS (2011). Gene expression signature-based prognostic risk score in gastric cancer. Clin Cancer Res.

[19] D'Errico M, de Rinaldia E, Blasi MF, Viti V, Falchetti M, Calcagnile A, Sera F, Saieva C, Ottini L, Palli D, Palombo F, Giuliani A, Dogliotti E (2009). Genome-wide expression profile of sporadic gastric cancers with microsatellite instability. Eur J Cancer.

[20] Nettl PA, Berk DA, Swatz MA, Grodzinsky AJ, Jain RK (2000). Role of extracellular matrix assembly in interstitial transport in solid tumors. Cancer Res.

[21] Brown EB, Boucher Y, Nasser S, Jaln RK (2004). Measurement of macromolecular diffusion coefficients in human tumors. Microvasc Res.

[22] Belin S, Beghin A, Solano-Gonzalez E, Bezin L, Brunet-Manquat S, Textoris J, Prats A, Mertani H, Dumontet C, Diaz J (2009). Dysregulation of ribosome biogenesis and translational capacity is associated with tumor progression of human breast cancer cells. PLoS One.

[23] Silvera D, Formenti SC, Schneider RJ (2010). Translational control in cancer. Nat Rev Cancer.

[24] Lamberti A, Caraglia M, Longo O, Marra M, Abbruzzese A, Arcari P (2004). The translation elongation factor 1A in tumorigenesis, signal transduction and apoptosis: review article. Amino Acids.

[25] Mémin E1, Hoque M, Jain MR, Heller DS, Li H, Cracchiolo B, Hanauske-Abel HM, Pe'ery T, Mathews MB (2014). Blocking eIF5A modification in cervical cancer cells alters the expression of cancer-related genes and suppresses cell proliferation. Cancer Res.

[26] Pino I, Pio R, Toledo G, Zabalegui N, Vicent S, Rey N, Lozano D, Torre W, Montuenga LM (2003). Altered patterns of expression of members of the heterogeneous nuclear ribonucleoprotein (hnRNP) family in lung cancer. Lung Cancer.

[27] Li S, Xu H, Ding H, Huang Y, Cao X, Yang G, Li J, Xie Z, Meng Y, Li X, Zhao Q, Shen B, Shao N (2009). Identification of an aptamer targeting hnRNP A1 by tissue slidebased SELEX. J Pathol.

[28] Fukukawa C, Ueda K, Nishidate T, Katagiri T, Nakamura Y (2010). Critical roles of LGN/GPSM2 phosphorylation by PBK/TOPK in cell division of breast cancer cells. Genes Chromosomes Cancer.

[29] Wang JJ, Gang ZX, Ye HM, You P, Cai MJ, Daun HB, Wang F, Zhang ZY (2013). Clinical significance of overexpressed cyclin-dependent kinase subunits 1 and 2 in esophageal carcinoma. Dis Esophaqus.

[30] Shen DY, Zhan YH, Wang QM, Rui G, Zhang ZM (2013). Oncogenic poteintial of cyclin kinase subunit-2 in cholagiocarcinoma. Liver Int.

[31] Chen L, Madura K (2005). Increased proteasome activity, ubiquitin-conjugating enzymes, and eEF1A translation factor detected in breast cancer tissue. Cancer Res.

[32] Bazzaro M, Lee MK, Zoso A, Stirling WL, Santillan A, Shih IM, Roden RB (2006). Ubiqutin-proteasome system stress sensitizes ovarian cancer to proteasome inhibitor-induced apoptosis. Cancer Res.

[33] Dimmeler S, Breitschopf K, Haendeler J, Zeiher AM (1999). Dephosphorylation targets Bcl-2 for ubiquitin-dependent degradation: a link between the apoptosome and the proteasome pathway. J Exp Med.

[34] Kumatori A, Tanaka K, Inamura N, Sone S, Ogura T, Matsumoto T, Tachikawa T, Shin S, Ichihara A (1990). Abnormally high expression of proteasomes in human leukemic cells. Proc Natl Acad Sci U S A.

[35] Kanayama H, Tanaka K, Aki M, Kagawa S, Miyaji H, Satoh M, Okada F, Sato S, Shimbara N, Ichihara A (1991). Changes in expressions of proteasome and ubiquitin genes in human renal cancer cells. Cancer Res.

[36] Ayllon V, O'Connor R (2007). PBK/TOPK promotes tumour cell proliferation through p38 MAPK activity and regulation of the DNA damage response. Oncogene.

[37] Hu F, Gartenhaus RB, Eichberg D, Liu Z, Fang HB, Rapoport AP (2010). PBK/TOPK interacts with the DBD domain of tumor suppressor p53 and modulates expression of transcriptional targets including p21. Oncogene.

[38] Zhu F, Zykova TA, Kang BS, Wang Z, Ebeling MC, Abe Y, Ma WY, Bode AM, Dong Z (2007). Bidirectional signals transduced by TOPK-ERK interaction increase tumorigenesis of HCT116 colorectal cancer cells. Gastroenterology.

[39] Keely PJ, Westwick JK, Whitehead IP, Der CJ, Parise LV (1997). Cdc42 and Rac1 induce integrin-mediated cell motility and invasiveness through PI(3)K. Nature.

[40] Zykova TA, Zhu F, Lu C, Higgins L, Tatsumi Y, Abe Y, Bode AM, Dong Z (2006). Lymphokine-activated killer T-cell-originated protein kinase phosphorylation of histone H2AX prevents arsenite-induced apoptosis in RPMI7951 melanoma cells. Clin Cancer Res.

[41] Dhillon AS, Hagan S, Rath O, Kolch W (2007). MAP kinase signaling pathway in cancer. Oncogene.

[42] Geng F, Wenzel S, Tanswy WP (2012). Ubiquitin and proteasomes in transcription. Annu Rev Biochem.

[43] O'Hara A, Howarth A, Varro A, Dimaline R (2013). The role of proteasome beta subunits in gastrin-mediated transcription of plasminogen activator inhibitor-2 and regenerating protein1. Plos one.

[44] Park JH, Lin ML, Nishidate T, Nakamura Y, Katagiri T (2006). PSZ-binding kinase/T-LAK cell-originated protein kinase, a putative cancer/testis antigen with an oncogenic activity in breast cancer. Cancer Res.

[45] Lei B, Qi W, Zhao Y, Li Y, Liu S, Xu X, Zhi C, Wan L, Shen H (2015). PBK/TOPK expression correlates with mutant p53 and affects patients' prognosis and cell proliferation and viability in lung adenocarcinoma. Hum Pathol.

