# Supplementary material for: PSMB8 and PBK as potential gastric cancer subtype-specific biomarkers associated with prognosis
Source: Oncotarget. 2016 Feb 15;7(16):21454–68. doi: 10.18632/oncotarget.7411 (PMC5008298; doi:10.18632/oncotarget.7411)
Supplement: Supplementary file 1 [file oncotarget-07-21454-s001.pdf]

PSMB8 and PBK as potential gastric cancer subtype-specific biomarkers associated with prognosis

Supplemetaty Material

Supplementary Table 1. Clinical characteristics of cancer tissue samples

characteristics		PNUH (n = 24)	CNUH (n = 24)
Age			
	≤ 65	12	7
	> 65	12	17
Sex			
	Male	15	20
	Female	9	4
Histologic type			
	Intestinal	17	18
	Diffuse	7	3
	Mixed		3
T stage			
	T1	1	1
	T2	3	6
	T3	15	9
	T4	5	8
Node stage			
	N0	4	6
	N1	4	7
	N2	6	5
	N3	10	6

Supplementary Table 2. Characteristics of highly expressed genes in each subtypes.

Category	Term	Genes	*P* value
highly expressed genes in C1			
GOTERM_BP_FAT	GO:0007155~cell adhesion	AEBP1, EPDR1, IGFBP7, TNC, CLDN5, COL3A1, FERMT2, SPOCK1, CDH5, ISLR, LAMB2, COL7A1, CD93, CTGF, COL6A3, COL6A1, ESAM, COL8A1, SPON1, THBS4, CYR61, COL18A1, SVEP1, PDPN, MGP, CPXM2, MFGE8, PCDH17, CERCAM, SIRPA, THY1, VWF, LAMA4, CD34, LAMA5, ITGA5, PECAM1, ROR2, VCAN, SGCE, ANTXR1, TGFB1I1, LAMC1, MFAP4, SELE, FEZ1	8.6E-18
GOTERM_BP_FAT	GO:0007010~cytoskeleton organization	CRYAB, CALD1, SIPA1, FSCN1, FERMT2, PDLIM3, MLH1, EVL, THY1, ARHGAP4, PFN2, LAMA5, SVIL, MYH11, SYNM, ANTXR1, CNN1, FGD5, ARAP3, DBN1, MYH10	5.4E-06
GOTERM_BP_FAT	GO:0001944~vasculature development	COL18A1, ACVRL1, PDPN, COL3A1, PRRX1, PRRX2, CDH5, THY1, LAMA4, BGN, LAMA5, CTGF, TGM2, SOX18, CYR61	1.7E-05
GOTERM_BP_FAT	GO:0051270~regulation of cell motion	COL18A1, ACVRL1, PDPN, MMP9, CHST3, GREM1, THY1, LAMA4, SERPINE2, LAMA5, PDGFRA, PDGFRB, ARAP3, IGFBP5	4.7E-06
KEGG_PATHWAY	hsa04512:ECM-receptor interaction	COL4A1, TNC, COL3A1, COL5A2, VWF, LAMA4, LAMB2, LAMA5, ITGA5, COL6A3, COL6A1, LAMC1, THBS4	2.4E-09
KEGG_PATHWAY	hsa04510:Focal adhesion	COL4A1, TNC, COL3A1, COL5A2, MYL9, VWF, LAMA4, LAMB2, ITGA5, LAMA5, COL6A3, PDGFRA, COL6A1, PDGFRB, LAMC1, MYLK, THBS4	2.6E-08
highly expressed genes in C2			
GOTERM_BP_FAT	GO:0006412~translation	EEF1B2, RPL6, RPLP0, RPL8, RPS6P1, EIF5A, LOC647030, RPS7, RPL29	5.6E-04
GOTERM_BP_FAT	GO:0006405~RNA export from nucleus	LOC399804, LOC645691, EIF5A, THOC4, HNRNPA1, THOC3	2.9E-05
GOTERM_BP_FAT	GO:0051436~negative regulation of ubiquitin-protein ligase activity during mitotic cell cycle	MAD2L1, PSMA6, BUB3, PSMB8	2.8E-03
GOTERM_BP_FAT	GO:0007049~cell cycle	LOC399804, MAEA, MAD2L1, PSMA6, CKS2, PBK, BUB3, PSMB8, CTNNB1	1.9E-02
KEGG_PATHWAY	hsa03010:Ribosome	RPL6, RPLP0, RPL8, RPS6P1, RPS7, RPL29	2.1E-04

Supplementary Table 3. Multivariate survival analysis of PSMB nuclear expression with Cox regression model in 385 patients with gastric cancer

Variables	B	SE	HR (95% CI)	*P*
Age (<60 and ≥60)	-0.169	0.294	0.845 (0.475–1.503)	0.566
Lymph node metastasis (absent vs. present)	-1.716	0.566	0.180 (0.059–0.545)	0.002
Gender (male vs. female)	0.142	0.291	1.152 (0.651–2.040)	0.627
Depth of invasion (T1 vs. T2, T3,T4)	-2.220	0.764	0.109 (0.024-0.485)	0.004
PSMB Nuclear expression (<5% and ≥5%)	-1.032	0.599	0.356 (0.110–1.153)	0.085

Note: B, coefficient; HR, hazard ratio; CI, confidence interval

Supplementary Table 4. Multivariate survival analysis of nuclear PBK expression with Cox regression model in 385 patients with gastric cancer

Variables	B	SE	HR (95% CI)	*P*
Age (<60 and ≥60)	-0.272	0.305	0.762 (0.420–1.384)	0.372
Lymph node metastasis (absent vs. present)	-1.714	0.563	0.180 (0.060–0.543)	0.002
Gender (male vs. female)	0.096	0.293	1.101 (0.620–1.954)	0.742
Depth of invasion (T1 vs. T2, T3,T4)	-2.250	0.761	0.105 (0.024-0.468)	0.003
PBK Nuclear expression (<5% and ≥5%)	-.852	0.490	0.427 (0.163–1.116)	0.083

Note: B, coefficient; HR, hazard ratio; CI, confidence interval

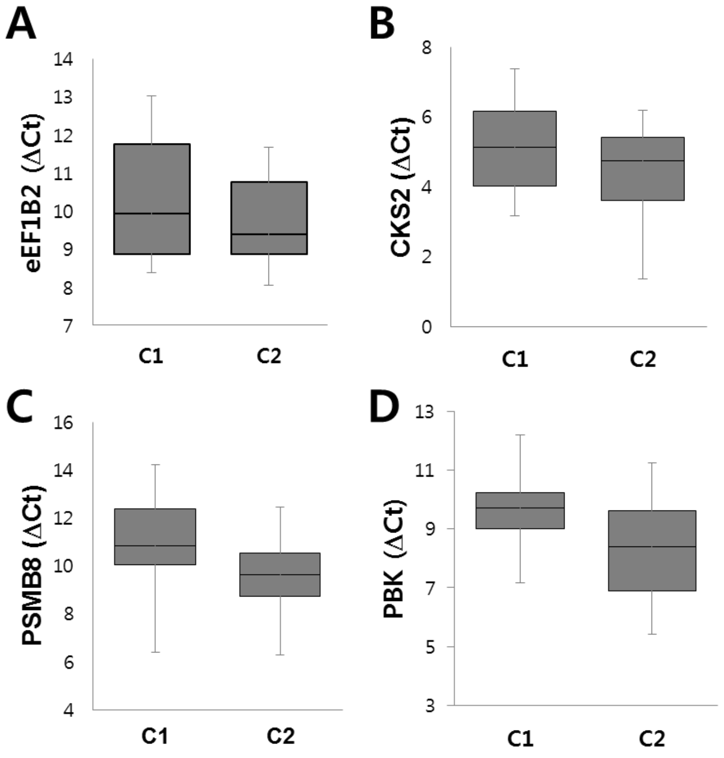

Supplementary Figure 1. Validation of highly expressed genes in cluster 2. eEF1B2 (**A**), CKS2 (**B**), PSMB8 (**C**) and PBK**(D)** expression were verified using real-time RT-PCR. Data were normalized to GAPDH, and mRNA abundance was calculated using the $2^{-\Delta\Delta CT}$ method.

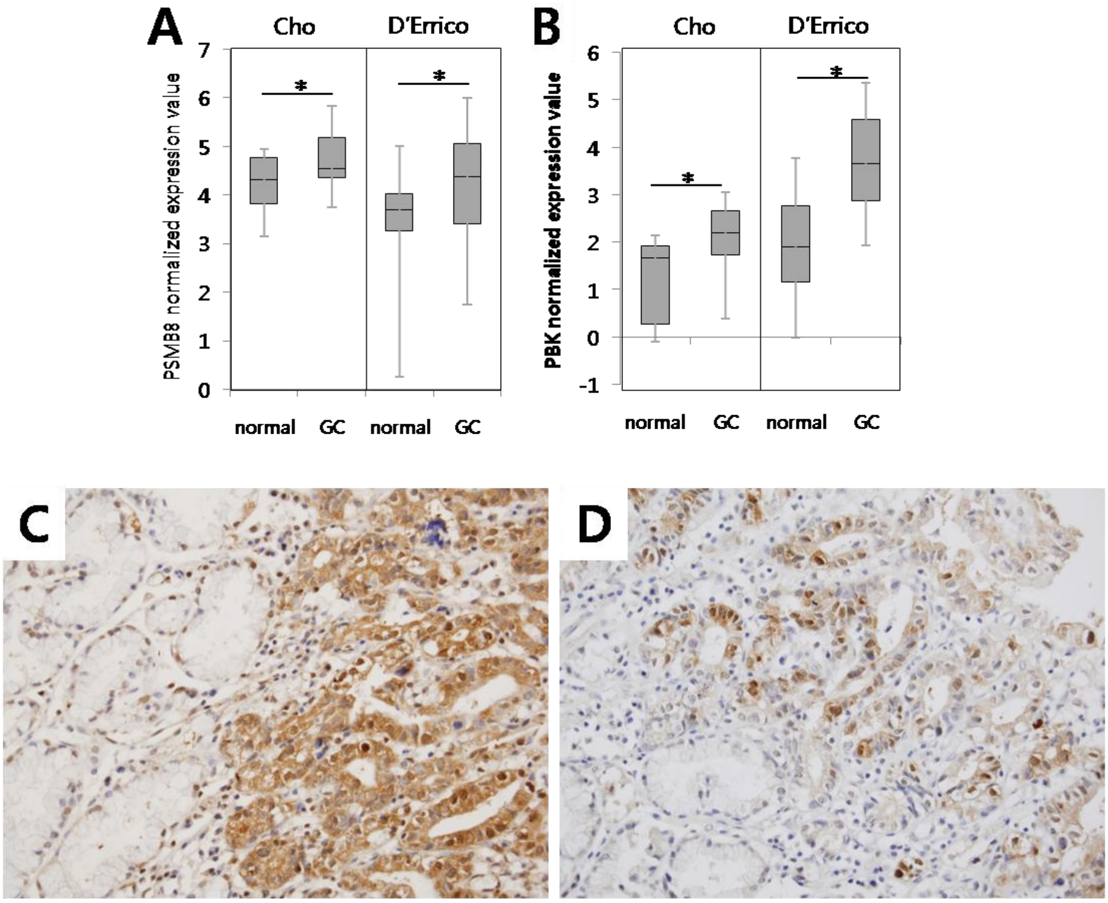

Supplementary Figure 2. Expression of PSMB8 and PBK in gastric cancer (GC) tissues and the adjacent normal tissues. The mRNA expression of PSMB8 (A) and PBK (B) in microarray datasets (Cho *et al*. and D'Errico *et al*.) that obtained from Oncomine database. ∗ $P < 0.05$. The protein expression of PSMB8 (C) and PBK (D) were determined using immunohistochemistry.

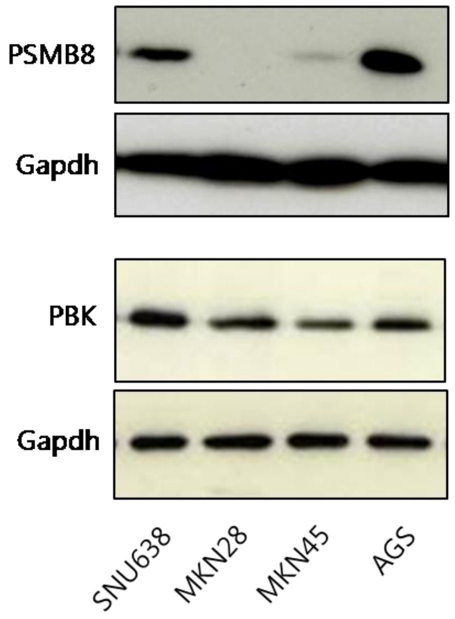

Supplementaty Figure 3. Expression of PSMB8 and PBK in gastric cancer cell lines. PSMB8 and PBK protein expression in gastric cancer cell lines (SNU638, MKN28, MKN45, and AGS) were determined using western blotting.

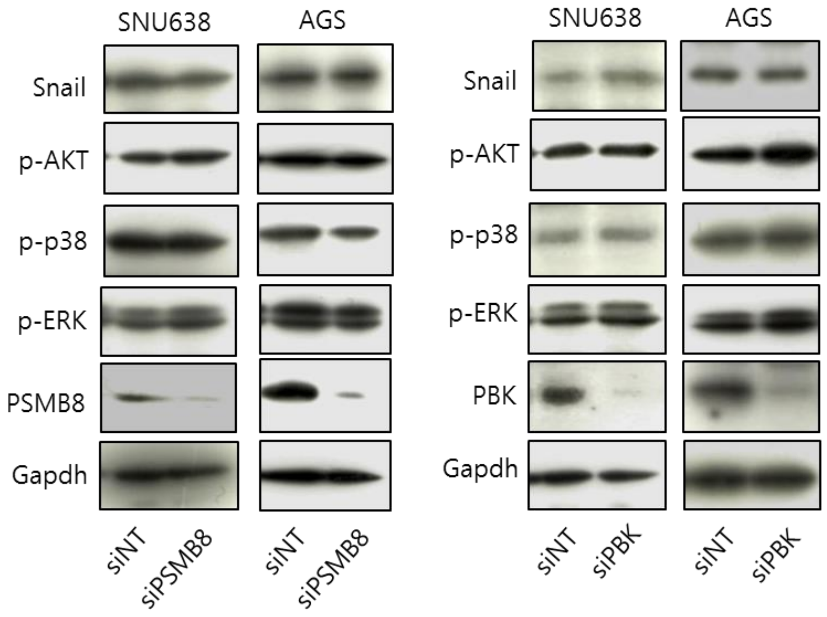

Supplementary Figure 4. Mechanisms underlying PSMB8 and PBK. SNU638 and AGS cells were transfected with either non-targeting siRNA (siNT), PSMB8 siRNA (siPSMB8), or PBK siRNA (siPBK). The expression of Snail, phospho-Akt, phospho-p38 MAPK, and phospho-ERK was measured by Western blotting.
